# Extracellular Vesicles from MSC Modulate the Immune Response to Renal Allografts in a MHC Disparate Rat Model

**DOI:** 10.1155/2015/486141

**Published:** 2015-08-13

**Authors:** M. Koch, A. Lemke, C. Lange

**Affiliations:** ^1^Department of Hepatobiliary Surgery and Transplantation, University Medical Center Hamburg-Eppendorf, 20246 Hamburg, Germany; ^2^Pediatric Nephrology, University Medical Center Hamburg-Eppendorf, 20246 Hamburg, Germany; ^3^Clinic for Stem Cell Transplantation, Department of Cell and Gene Therapy, University Medical Center Hamburg-Eppendorf, 20246 Hamburg, Germany

## Abstract

Application of mesenchymal stromal cells (MSC) has been proposed for solid organ transplantation based on their potent immunomodulatory effects. Since side effects from the injection of large cells cannot be excluded, the hypothesis rises that extracellular vesicles (EV) may cause immunomodulatory effects comparable to MSC without additional side effects. We used MSC-derived EV in a rat renal transplant model for acute rejection. We analysed peripheral blood leukocytes (PBL), kidney function, graft infiltrating cells, cytokines in the graft, and alloantibody development in animals without (allo) and with EV application (allo EV). There was no difference in kidney function and in the PBL subpopulation including Tregs between allo and allo EV. In the grafts T- and B-cell numbers were significantly higher and NK-cells lower in the allo EV kidneys compared to allo. TNF-*α* transcription was lower in allo EV grafts compared to allo; there was no difference regarding IL-10 and in the development of alloantibodies. In conclusion, the different cell infiltrates and cytokine transcription suggest distinct immunomodulatory properties of EV in allotransplantation. While the increased T- and B-cells in the allo EV grafts may represent a missing or negative effect on the adaptive immune system, EV seem to influence the innate immune system in a contrary fashion.

## 1. Introduction

Life-long drug-based immunosuppression still is the standard regime to induce clinical allograft acceptance, however, at the price of significantly reduced overall well-being of transplanted patients. Therefore, research into alternative treatment approaches is warranted to decrease the need for immunosuppressive medication, improve long-term graft survival, and ideally induce tolerance. In the highly complex pathophysiology of acute renal allograft rejection, several components of the immune system are involved leading to vascular, glomerular, and tubular injuries. While pharmacological interventions often target one aspect only, cell-based therapies have the potential to influence multiple pathophysiological mechanisms.

Mesenchymal stromal cells (MSC) are of special therapeutic interest because of their capacity to enhance tissue repair by secreting bioactive molecules that (a) inhibit apoptosis and limit the extent of damage or injury, (b) inhibit fibrosis or scarring at sites of injury, (c) protect the microvasculature and stimulate angiogenesis to improve perfusion, and (d) stimulate the mitogenesis of tissue-intrinsic progenitor cells [1–4]. Additionally, MSC may play specific roles as modulators in the maintenance of peripheral and transplantation tolerance, autoimmunity, tumor evasion, and fetal-maternal tolerance [4, 5]. MSC influence all components of the immune system as shown for T-, B-, natural killer- (NK-), monocytic and dendritic cells* in vitro* and* in vivo *[6, 7]. Nevertheless the application of MSC in solid organ transplantation (SOT) has shown some hurdles regarding safety in several studies. Our own group and others provided evidence for a negative impact on recipient survival in rat models of acute kidney allograft rejection [8, 9] and several questions have been rising about the role of MSC in SOT [10].

Recently, extracellular vesicles (EV) have been shown to play important roles in intercellular communications [11–14]. EV generated from MSC might have potent immunomodulatory properties similar to their parental cells, but missing potential side effects of the application of large cells. It has been shown that several effects of MSC are transmitted via EV [15, 16] and that EV are effective in models of acute kidney injury [17, 18] as described before for MSC [19–21].

Very few studies of EV from different sources are available in SOT in animal models [22, 23] demonstrating a tolerogenic effect via MHC class II presentation.

The aim of our study was to analyze the effect of recipient derived EV from MSC on allograft rejection in a rat renal transplant model with exclusive and complete MHC disparity.

## 2. Materials and Methods

### 2.1. Mesenchymal Stromal Cells (MSC) and Microvesicles (EV)

In this experimental setting, we used isogeneic MSC. Bone marrow was procured from Lewis (LEW) rats (recipient strain) by flushing femurs and tibiae. Cells were resuspended in DMEM/Hams-F12 medium supplemented with 20% preselected fetal bovine serum (both, Biochrom, Germany) and 2 mol/L L-glutamine (Gibco, Germany) and seeded in tissue culture flasks (Greiner, Germany). Plastic adherent cells were grown to near confluency, passaged, and stored in liquid nitrogen as passages 3-4 and used as working cell bank. Expanded MSC were characterized for their phenotype using flow cytometry and differentiation capability into adipogenic, osteogenic, and chondrogenic lineages as described previously [9, 24]. Cells of passages 7–9 were used throughout all transplantation experiments. No antibiotics were used for cell expansion to avoid sublevel microbial contamination. Regular testing for mycoplasma was performed.

Expanded MSC at a confluency of 80% were fed with medium containing EV-depleted FCS (ultracentrifugation for 12 hours at 4°C with 100,000 g). Thereafter, MSC were cultured for 16 hours in DMEM/Hams-F12 medium supplemented with 0.5% EV-depleted bovine serum albumin (Sigma, Germany) and 2 mol/L L-glutamine. EV from the supernatant were collected after depletion of cells and cell debris by centrifugation for 20 min at 4°C with 2,000 g, depletion of apoptotic bodies for 20 min at 4°C with 12,000 g, and concentration by ultracentrifugation for 70 min at 4°C with 100,000 g with swinging buckets in a final volume of 1 mL in PBS. The MSC from the expansion cell cultures were counted with Trypan blue and the amount of EV correlated to the cell number from which the EV were harvested.

### 2.2. Kidney Transplantation

Animal experiments were approved by The Local Ethical Committee (number 49/09) and performed according to local and EU guidelines. Male LEW rats (LEW, RT1^l^) (Charles River, Germany) received a kidney graft from weight and age matched LEW.1U rats (LEW.1U, RT1^u^) (Zentrales Tierlabor, Medizinische Hochschule Hannover, Germany). Sharing the same genetic background, donor and recipient differed completely in MHC haplotypes, resulting in MHC class I (RT1.A and RT1.C) as well as MHC class II (RT1.B/D) incompatibilities [25].

Life-sustaining RTx was performed as previously described [26]. Graft ischemia time was limited to 30 minutes. The left kidney of the recipient was removed during transplantation whereas the right kidney was excised 5 days after transplantation. All animals were harvested on day 7 after transplantation.

As a control group, rats received a kidney graft from the same strain (isogeneic control (iso) group; LEW → LEW, *n* = 3).

After a fully MHC-mismatched kidney transplantation (LEW.1U → LEW) rats received either medium (allo, *n* = 6) or EV (allo EV, *n* = 7) on day 1 after transplantation. We included only animals in the experiment with an adequate general condition on day 1 after transplantation.

### 2.3. Renal Function Assays

Serum creatinine (SCr) was analyzed after nephrectomy and on day 7 with Reflovet Plus (Roche Diagnostics, Switzerland; detection limit 0.5 mg/dL). Body weight and general condition were monitored daily.

### 2.4. Flow Cytometry Analysis of Peripheral Blood Lymphocytes

Peripheral blood lymphocytes were stained with the following antibodies (all, Biolegend, San Diego, California, USA): CD3 (1F4), CD4 (W3/25), CD8 (Ox-8), CD45 (Ox-1), CD161 (10/78), CD25 (Ox-39), FoxP3 (150D), CD45RA (Ox-33), and polyclonal Goat anti-Rat IgG and IgM (Dianova, Hamburg, Germany).

Peripheral blood lymphocytes were obtained by treatment of EDTA blood samples with commercially available erythrocyte lysis buffer (Ortho Diagnostics, Neckargemuend, Germany). Samples of 0.5–1.0 × 10^6^ cells were washed twice and incubated with 50 *μ*L fluorescence labeled primary antibody for 30 min at 4°C in the dark. Intracellular staining for regulatory T-cells (Tregs) was performed with an Alexa Fluor 647 antihuman FoxP3 Flow Kit (206D, Biolegend, San Diego, CA) according to the manufacturer's guidelines in a triple staining with CD25 and CD4. After surface staining with CD25 and CD4, cells were fixed, permeabilized, and stained with FoxP3.

Fluorescence analysis was performed on a FACScanto (BD Bioscience, San Jose, California, USA). 1 × 10^4^ cells measured with a standardized lymphocyte live gate were accumulated on logarithmic scales and analyzed using a FlowJo computer program (Ashland, Orlando, USA).

Differential blood counts were performed to calculate the numbers of lymphocyte subpopulation per *μ*L.

### 2.5. Histopathology

For immunohistochemistry on frozen sections, the following mAbs were used: R73 (rat TCR constant determinant; Biolegend, San Diego, California, USA), ED1 (rat tissue macrophages, monocytes, and dendritic cells), 10/78 (CD161, NK-cells) (both, Serotec, Germany), Ki-B1R (rat pan B-cell marker; Dianova, Hamburg, Germany), and 3.4.1 (CD8, BD Biosciences, San Jose, California, USA). Single staining techniques were performed as described previously [27]. Briefly, 5 *μ*m sections were blocked, incubated with primary antibody, washed, and treated with peroxidase-coupled rat-anti-mouse IgG (Dianova, Hamburg, Germany). Peroxidase activity was visualized with 3-amino-9-ethyl-carbazole. Sections were counterstained with Mayer's Hemalaun (Merck, Germany).

Graft infiltrating cells within the renal cortex were counted in ten 400-fold high power fields (hpf) per section. Five representative animals of each group were analyzed for graft infiltrating cells by an independent investigator.

### 2.6. Gene Expression Examination with Quantitative RT-PCR

Total RNA from harvested kidneys was isolated and purified using RNeasy Mini Kit (Qiagen, Germany). Quantity and quality of RNA were determined using Infinity M200 (Tecan, Germany). Complementary DNA was synthesized using SuperScript III Reverse Transcriptase (Invitrogen, Germany).

Quantitative real-time PCR (qRT-PCR) for interleukin-10 (IL-10) and tumor necrosis factor-alpha (TNF*α*) were performed on a Thermocycler MX3000P (Stratagene, Germany) using QuantiFast SYBR Green PCR Kit (Qiagen, Germany) or SYBR Premix Ex Taq (Lonza, Switzerland) for Gapdh with the following primers: QT00177618 for IL-10 (Qiagen, Germany) and QT00178717 (Qiagen, Germany) for TNF*α*. Gene transcriptions were normalized to Gapdh (QT00199633; Qiagen). The mRNA expression level was calculated by the ΔΔCt method in comparison to iso (*n* = 3).

Representative samples of renal cortex of each group were used: iso *n* = 3, allo *n* = 5, allo EV *n* = 6.

### 2.7. Detection of MHC Antibodies

To analyze the presence of circulating donor-specific anti-MHC antibodies, sera of transplanted animals were incubated with peripheral blood lymphocytes of donor rat strain (LEW.1U) and recipient strain (LEW) as a control as described previously [25]. In short, after incubation with recipients sera lymphocytes were double stained for rat immunoglobulins and CD4 (mAbw3/25). T-cells become positive for rat IgG if anti-MHC class I antibodies are present in recipient's sera. The difference of the mean fluorescence intensity (MFI) of T-cells from LEW.1U and LEW rats is given as ΔMFI for time point d0 (before transplant) and d7 (after transplant).

Samples were analyzed using a FACScanto (BD Bioscience, San Jose, California, USA) and results were evaluated using FlowJo computer program (Ashland, Orlando, USA).

### 2.8. Statistical Analysis

Statistical analyses were performed using GraphPad Prism version 6.04 for Windows, (GraphPad Software, USA). Unpaired two-tailed *t*-test was used to compare creatinine values and numbers of graft infiltrating cells between allo and allo EV group. Nonparametric Mann-Whitney test was used to compare intragraft TNF*α* and Il-10 expression.

## 3. Results

### 3.1. Kidney Function in Allo Groups Is Impaired

**Figure 1:**
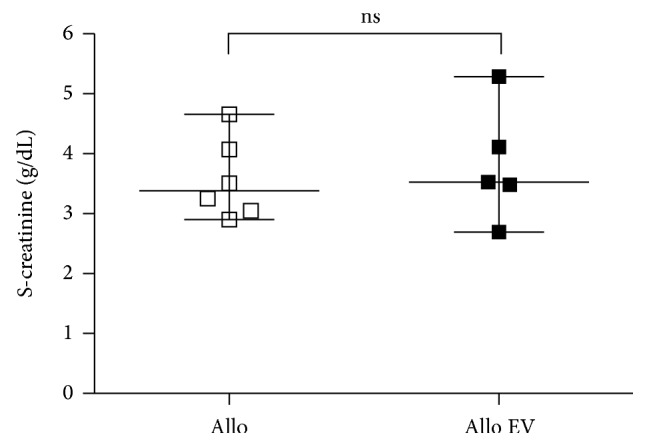
Serum creatinine (SCr) at day 7 after transplantation. There was no significant difference between the allo and the allo EV group.

In both allo groups we observed a severely impaired kidney function. SCr in the allo groups on day 7 was not different in animals with and without EV application. The mean SCr in the allo group was 3.6 ± 0.7 g/dL and was 3.8 ± 1.0 g/dL (*p* = 0.63) in the allo EV group (Figure 1).

### 3.2. Peripheral Blood Mononuclear Cells Were Not Different between Groups

Analyzing the peripheral blood cell composition of transplanted animals on day 7 after transplantation, no difference in the percentage of CD3+/CD4+ T-cells was detected. CD4+/CD25+ cells were also not different (both not shown). CD4+/FoxP3+ regulatory T-cells were decreased in both allo groups compared to the iso animals, however, without reaching statistically significant difference between the allo and the allo EV group (data not shown). The number of B-cells in the allo EV group was lower compared to allo animals (not shown), however, at a nonsignificant level. CD8 T-cells and NK-cells were slightly increased in the allo EV group, not reaching statistical significance, too.

### 3.3. EV Injection Modulates Lymphocyte Infiltration in Grafts

**Figure 2:**
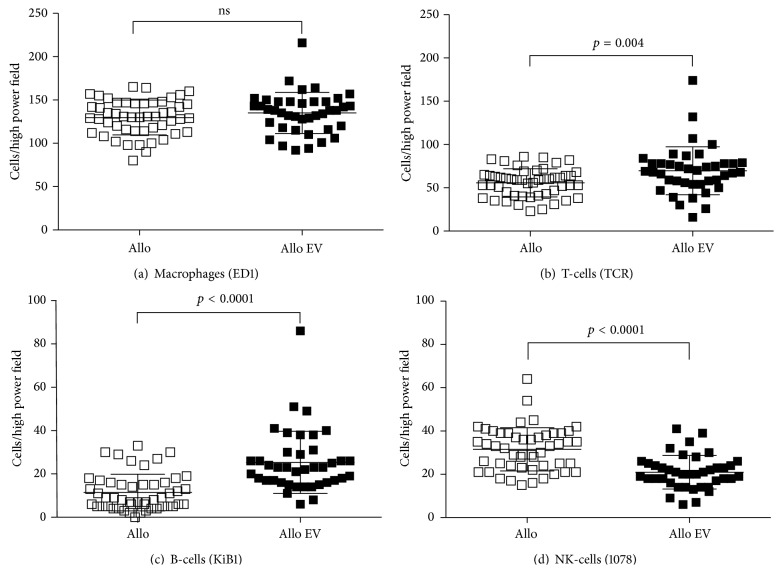
Cell infiltrates in kidney grafts. Graft infiltrates of macrophages ((a): ED1), T-cells ((b): TCR), B-cells ((c): KiB1), and NK-cells ((d): 10/78) in the allo group compared to allo EV as cells per high power field. While macrophages were not different (a), T- and B- cells ((b), (c)) were significantly more frequent in kidneys from the allo EV group while NK-cells were reduced compared to allo (d).

In kidney grafts harvested on day 7 after transplantation, there was a massive cell infiltrate of mononuclear cells in both allo groups while there were only very few infiltrating lymphocytes in the iso group (data not shown). In allotransplanted grafts macrophages and T-cells were the most prominent infiltrating cells. No difference regarding the infiltration of macrophages (Figure 2(a)) in the allo EV and the allo group has been observed. The number of T- and B-cells was higher in the allo EV animals compared to allo (Figures 2(b) and 2(c), *p* = 0.004 and *p* < 0.0001 for T- and B-cells, resp.), while NK-cell infiltrates were reduced (Figure 2(d), *p* < 0.0001).

### 3.4. Intragraft TNF*α* and IL-10 Expression

**Figure 3:**
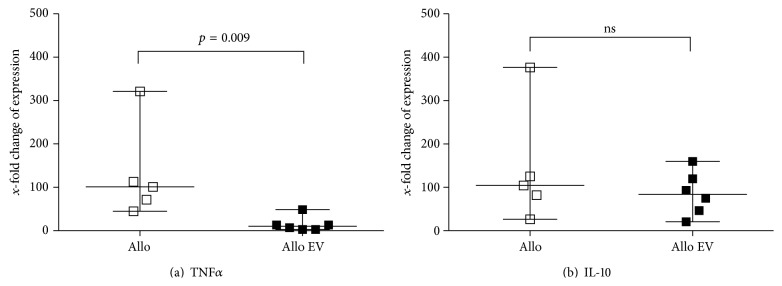
Gene expression in transplanted kidney grafts (*x*-fold change compared to iso controls). TNF*α* (a) was significantly less expressed in allo EV compared to allo while IL-10 (b) was not significantly different between both groups.

Median TNF*α* expression in the allo group was 100.9-fold (range of 44.8–216.9) compared to iso transplanted animals as shown in Figure 3(a). TNF*α* was significantly less expressed in allo EV grafts (median 10.1, range of 2.6–48.7). In contrast, IL-10 did not differ between allo and allo EV kidney grafts (Figure 3(b)).

### 3.5. Development of MHC Antibodies

Sera of representative allo transplanted animals were tested for donor-specific MHC antibodies before transplantation and on day 7. While prior to transplantation (day 0) none of the sera were positive for antibodies against LEW.1U cells, and all tested sera became positive on day 7 (allo group *n* = 3, allo EV group *n* = 4). There was no difference regarding the mean fluorescence intensity (MFI, allo 1462 ± 885; allo EV 1439 ± 565) between the groups.

## 4. Discussion

This is the first report on the results of immunomodulation with MSC-derived EV in a renal transplantation model with exclusive and complete MHC disparity.

EV are known to be an essential mediator in cell-cell communication by horizontal transfer of lipids, proteins, mRNAs, and microRNAs [28]. In our experiments we wanted to take advantage of biomolecule-transfer via EV, thereby circumventing the lethal side effects previously observed with injection of MSC in the same model [8]. EV did not alter the animal survival and no side effects were observed. However, SCr in the allo EV group did not differ from the values in the allo group; that is, there was no difference in graft function, which was impaired due to acute rejection in both allo groups.

It has previously been shown that application of MSC and MSC-derived EV can ameliorate ischemia-reperfusion injury (IRI) [13, 15–17]. EV from other sources, for example, Wharton's Jelly, affected IRI by suppressing CX3CL1 [18]. In their model the authors described decreased levels of TNF*α* and increased IL-10 after EV injection. The reduction of TNF*α* is in line with our data, but we could not confirm an increase of IL-10. This might be due to the fact that we used an allotransplantation model where IL-10 was increased* per se*. Furthermore, we could not confirm a decrease of macrophages infiltrates in the grafts but again there is a mechanistical difference between IRI and allotransplantation.

The kidneys in our experiment were analyzed 7 days after transplantation. It is likely that at this time point IRI is already overruled by the adaptive alloimmune response. In allotransplantation, TNF*α* is regarded as a proinflammatory cytokine which is mainly produced by macrophages and NK-cells [29, 30]. The significant reduction of TNF*α* in the graft is in accordance with previous data describing immunomodulation by MSC [1]. It is possible that (1) EV interact with graft infiltrating macrophages resulting in lower cytokine production (the number of macrophages is not influenced in our model) and (2) that NK-cells, which are significantly reduced in the allo EV group, are the major source of TNF*α* in our model.

IL-10 as an anti-inflammatory or tolerogenic cytokine was significantly higher expressed in allo transplanted kidneys compared to the iso controls. But comparing both allo groups, there was no difference regarding IL-10 in the kidneys or Tregs in the blood of the animals. We need to admit that we did not stain Tregs in the grafts, which might be influenced differently in comparison to the peripheral blood lymphocytes.

*In vitro* data demonstrated a clear-cut modulation of T-cell activity by EV creating a regulatory phenotype [31, 32]. In our model, FoxP3 positive T-cells in the blood of the EV treated animals were not increased but in contrast were the lowest compared to iso and allo animals. T-cell infiltrates in the grafts of EV treated animals are even increased. Together with the data on IL-10 transcription in the grafts, our data do not support a tolerogenic effect of EV in kidney transplantation.

Studies are available describing an inhibitory effect of MSC and EV on B-cell activity [31]. In our study there was significantly higher number of B-cells infiltrating the transplanted grafts of EV treated animals. All animals tested developed antibodies against the donor within 7 days and there was no difference in the MFI as a relative marker for the amount of antibodies.

We propose that T- and B-cell activation in a strong reactive allotransplantation model cannot be ameliorated by EV, since MHC dependent mechanisms might be much stronger compared to nonimmunological injury in IRI.

It is remarkable that we saw a reduced NK-cell number and nearly no TNF*α* transcription in the EV treated grafts. This might lead to the hypothesis that EV influence more the innate than the adaptive immune system.

It needs to be discussed that we used recipient type (LEW) derived MSC for production of EV, while other authors used donor derived cells [23]. This might influence the tolerogenic capacities of the EV. Thinking of a clinical application, our approach might be more applicable compared to donor derived generation of EV. Recipient type MSC can be isolated and expanded at any time point from the recipient, while donor MSC are only available in living donation. Still, it cannot be ruled out that donor derived MSC or EV might cause sensitization of the recipient and it remains unclear if the immunological impact of donor MSC or EV is dependent on the source of cells. A second reason for the different outcomes compared to Pêche et al. might have to be sought in the methodological approach. In our model, the transplanted kidney had to become fully functional after removal of the second recipient kidney, whereas Pêche et al. used the heterotopic heart transplantation where the transplanted heart does not have to fulfill life-sustaining activity.

We conclude that EV can be administered safely in a rat renal transplantation model. No fatal side effects have been seen in the rats. There was no clinical difference regarding kidney function between the allo and the allo EV group. We could not prove an effect of EV on T- or B-cell mediated acute rejection, but we demonstrated different pattern of graft infiltrating lymphocytes and cytokines in the grafts induced by EV.

Suggesting a dominant effect of recipient derived EV on the innate immune system, but not an adequate suppression of the adaptive immunity, additional immunosuppression might be needed to induce significant modulation of allograft rejection with EV.

## Abbreviations

MSC: Mesenchymal stromal cells
EV: Extracellular vesicle
IRI: Ischemia-reperfusion injury
RTx: Renal transplantation
SCr: Serum creatinine
hpf: High power field.

## References

[1] Aggarwal S., Pittenger M. F. (2005). Human mesenchymal stem cells modulate allogeneic immune cell responses. *Blood*.

[2] Haynesworth S. E., Baber M. A., Caplan A. I. (1996). Cytokine expression by human marrow-derived mesenchymal progenitor cells in vitro: effects of dexamethasone and IL-1*α*. *Journal of Cellular Physiology*.

[3] Koç O. N., Lazarus H. M. (2001). Mesenchymal stem cells: heading into the clinic. *Bone Marrow Transplantation*.

[4] Uccelli A., Moretta L., Pistoia V. (2008). Mesenchymal stem cells in health and disease. *Nature Reviews Immunology*.

[5] Nauta A. J., Fibbe W. E. (2007). Immunomodulatory properties of mesenchymal stromal cells. *Blood*.

[6] Le Blanc K., Samuelsson H., Gustafsson B. (2007). Transplantation of mesenchymal stem cells to enhance engraftment of hematopoietic stem cells. *Leukemia*.

[7] Uccelli A., Moretta L., Pistoia V. (2006). Immunoregulatory function of mesenchymal stem cells. *European Journal of Immunology*.

[8] Koch M., Lehnhardt A., Hu X. (2013). Isogeneic MSC application in a rat model of acute renal allograft rejection modulates immune response but does not prolong allograft survival. *Transplant Immunology*.

[9] Seifert M., Stolk M., Polenz D., Volk H.-D. (2012). Detrimental effects of rat mesenchymal stromal cell pre-treatment in a model of acute kidney rejection. *Frontiers in Immunology*.

[10] Haarer J., Johnson C. L., Soeder Y., Dahlke M. H. (2015). Caveats of mesenchymal stem cell therapy in solid organ transplantation. *Transplant International*.

[11] Gould S. J., Raposo G. (2013). As we wait: coping with an imperfect nomenclature for extracellular vesicles. *Journal of Extracellular Vesicles*.

[12] Raposo G., Stoorvogel W. (2013). Extracellular vesicles: exosomes, microvesicles, and friends. *Journal of Cell Biology*.

[13] Théry C. (2011). Exosomes: secreted vesicles and intercellular communications. *F1000 Biology Reports*.

[14] Witwer K. W., Buzás E. I., Bemis L. T. (2013). Standardization of sample collection, isolation and analysis methods in extracellular vesicle research. *Journal of Extracellular Vesicles*.

[15] Akyurekli C., Le Y., Richardson R. B., Fergusson D., Tay J., Allan D. S. (2015). A systematic review of preclinical studies on the therapeutic potential of mesenchymal stromal cell-derived microvesicles. *Stem Cell Reviews and Reports*.

[16] György B., Hung M. E., Breakefield X. O., Leonard J. N. (2015). Therapeutic applications of extracellular vesicles: clinical promise and open questions. *Annual Review of Pharmacology and Toxicology*.

[17] de Almeida D. C., Donizetti-Oliveira C., Barbosa-Costa P., Origassa C. S., Camara N. O. (2013). In search of mechanisms associated with mesenchymal stem cell-based therapies for acute kidney injury. *The Clinical Biochemist Reviews*.

[18] Zou X., Zhang G., Cheng Z. (2014). Microvesicles derived from human Wharton's Jelly mesenchymal stromal cells ameliorate renal ischemia-reperfusion injury in rats by suppressing CX3CL1. *Stem Cell Research & Therapy*.

[19] Lange C., Tögel F., Ittrich H. (2005). Administered mesenchymal stem cells enhance recovery from ischemia/reperfusion-induced acute renal failure in rats. *Kidney International*.

[20] Tögel F., Hu Z., Weiss K., Isaac J., Lange C., Westenfelder C. (2005). Administered mesenchymal stem cells protect against ischemic acute renal failure through differentiation-independent mechanisms. *The American Journal of Physiology—Renal Physiology*.

[21] Tögel F. E., Westenfelder C. (2010). Mesenchymal stem cells: a new therapeutic tool for AKI. *Nature Reviews Nephrology*.

[22] Pêche H., Heslan M., Usal C., Amigorena S., Cuturi M. C. (2003). Presentation of donor major histocompatibility complex antigens by bone marrow dendritic cell-derived exosomes modulates allograft rejection. *Transplantation*.

[23] Pêche H., Renaudin K., Beriou G., Merieau E., Amigorena S., Cuturi M. C. (2006). Induction of tolerance by exosomes and short-term immunosuppression in a fully MHC-mismatched rat cardiac allograft model. *American Journal of Transplantation*.

[24] Jaquet K., Krause K. T., Denschel J. (2005). Reduction of myocardial scar size after implantation of mesenchymal stem cells in rats: what is the mechanism?. *Stem Cells and Development*.

[25] Poehnert D., Broecker V., Mengel M., Nashan B., Koch M. (2010). Induction of chronic renal allograft dysfunction in a rat model with complete and exclusive MHC incompatibility. *Transplant Immunology*.

[26] Koch A., Joosten S. A., Mengel M., van Kooten C., Paul L. C., Nashan B. (2005). Adoptive transfer of primed CD4^+^ T-lymphocytes induces pattern of chronic allograft nephropathy in a nude rat model. *Transplantation*.

[27] Doege C., Koch M., Heratizadeh A., Sótonyi P., Mengel M., Nashan B. (2005). Chronic allograft nephropathy in athymic nude rats after adoptive transfer of primed T lymphocytes. *Transplant International*.

[28] Ratajczak M. Z., Kucia M., Jadczyk T. (2012). Pivotal role of paracrine effects in stem cell therapies in regenerative medicine: can we translate stem cell-secreted paracrine factors and microvesicles into better therapeutic strategies. *Leukemia*.

[29] Caron G., Delneste Y., Aubry J.-P. (1999). Human NK cells constitutively express membrane TNF-*α* (mTNF*α*) and present mTNF*α*-dependent cytotoxic activity. *European Journal of Immunology*.

[30] Fehniger T. A., Shah M. H., Turner M. J. (1999). Differential cytokine and chemokine gene expression by human NK cells following activation with IL-18 or IL-15 in combination with IL-12: implications for the innate immune response. *Journal of Immunology*.

[31] Conforti A., Scarsella M., Starc N. (2014). Microvescicles derived from mesenchymal stromal cells are not as effective as their cellular counterpart in the ability to modulate immune responses in vitro. *Stem Cells and Development*.

[32] Favaro E., Carpanetto A., Lamorte S. (2014). Human mesenchymal stem cell-derived microvesicles modulate T cell response to islet antigen glutamic acid decarboxylase in patients with type 1 diabetes. *Diabetologia*.

